# Waveguide-Based Fluorescent Immunosensor for the Simultaneous Detection of Carbofuran and 3-Hydroxy-Carbofuran

**DOI:** 10.3390/bios10120191

**Published:** 2020-11-27

**Authors:** Weiming Sun, Lanhua Liu, Abdul Ghaffar Memon, Xiaohong Zhou, Hongwei Zhao

**Affiliations:** 1 Key Laboratory of A & F Environmental Processes and Ecological Regulation of Hainan Province, College of Ecology and Environment, Hainan University, Haikou 570228, China; 18095131210068@hainanu.edu.cn; 2 State Key Laboratory of Marine Resource Utilization in South China Sea, Hainan University, Haikou 570228, China; 3 State Key Joint Laboratory of ESPC, School of Environment, Tsinghua University, Beijing 100084, China; llh1987@mail.tsinghua.edu.cn (L.L.); abdulghaffar@neduet.edu.cn (A.G.M.); 4 Department of Environmental Engineering, NED University of Engineering and Technology, Karachi 75270, Pakistan

**Keywords:** carbofuran, 3-hydroxy-carbofuran, immunosensor, evanescent wave, antibody

## Abstract

Carbofuran (CBF) is an efficient and broad-spectrum insecticide. As testing indicators for water quality and agricultural products, CBF and its metabolite 3-hydroxy-carbofuran (3-OH-CBF) are regulated by many countries. The detection of CBF and 3-OH-CBF is of great importance for the environment and human health. However, an immunosensor detection method for the simultaneous analysis of CBF and 3-OH-CBF remains unavailable. Herein, we report a waveguide-based fluorescent immunosensor for detecting CBF and 3-OH-CBF, synchronously. The immunosensor is based on a broad-spectrum monoclonal antibody with high binding affinity against CBF and 3-OH-CBF. The linear detection ranges for CBF and 3-OH-CBF are 0.29–2.69 and 0.12–4.59 μg/L, with limits of detection of 0.13 μg/L for CBF and 0.06 μg/L for 3-OH-CBF, respectively. The whole detection process for each cycle is approximately 30 min. The results show a good application prospect for the rapid detection of CBF and 3-OH-CBF in water or agricultural products.

## 1. Introduction

Carbofuran (CBF) is a broad-spectrum and highly toxic insecticide [1]. Owing to its high toxicity and long environmental residual duration, it has been prohibited for use in several countries [2,3,4]. CBF and its metabolite 3-hydroxy-carbofuran (3-OH-CBF) block acetylcholinesterase’s function, which induces acetylcholine overaccumulation at neuromuscular junctions and creates nausea, vomiting, and other risks. Currently, however, CBF is still used in some countries and regions worldwide [5]. It has been reported that CBF is a common residual pesticide in agricultural products from all over the world [6,7,8].

CBF is easy to be degraded into 3-OH-CBF in the environment [9]. To prevent the risk caused by CBF, the acceptable daily intake of CBF is set to be 0.001 mg/kg by the Chinese government (2019), and the maximum residue limits (MRLs) of CBF and its metabolite 3-OH-CBF, which refers to the sum of CBF and 3-OH-CBF (tCBF), in various types of foods have been strictly restricted. For example, in China, the MRLs of tCBF in cereals, vegetables (except potatoes), potatoes, fruits, and meat are 1, 0.02, 0.1, 0.02, and 0.05 mg/kg, respectively. In the European Union, the MRLs of tCBF in apples, potatoes, lettuce, and citrus fruits are 0.001, 0.001, 0.002, and 0.01 mg/kg, respectively (EFSA, 2014). Apart from the agricultural products, the MRLs of CBF in drinking water are set to 0.007 mg/L in China and 0.04 mg/L in USA (US EPA, 2018), respectively.

Various analytical methods including gas chromatography (GC) [10,11,12], gas chromatography–mass spectrometry (GC–MS) [6,13], high performance liquid chromatography (HPLC) [14], and liquid chromatography–mass spectrometry (HPLC–MS) [15] have been reported for CBF monitoring. These methods are sensitive and reliable for quantitative analysis of CBF, however require expensive instruments, complicate pretreatment, and time-consuming procedures. Other technologies for CBF detection include enzyme-linked immunosorbent assay (ELISA) [16], immunoassay-based colloidal gold test strip (GICA) [17] and fluorimetry [18]. Notably, the immunosensor is a powerful technology widely used in the detection of CBF at trace concentrations by compositing sensitivity and simplicity. Up to now, numerous electrochemical [19,20,21,22] and other antibody-based [23] sensors have been developed. Exploring a novel immunosensor with different transducers for CBF detection is still highly demanded and worthy to be addressed.

Given that CBF can be partially metabolized in the environment into 3-OH-CBF, it is necessary to detect both CBF and 3-OH-CBF. Simultaneous detection of CBF and 3-OH-CBF is currently achieved primarily by using chromatography-based methods, however, their disadvantages have already been stated above. To address this issue, we previously have reported a broad-spectrum monoclonal antibody and developed the indirect competitive ELISA [24] and GICA [25] for simultaneous detection of CBF and 3-OH-CBF. ELISA-based analysis requires a relatively longer time of analysis, multi-step operating procedures, and skilled staff. The GICA test is rapid, simple, and easy to determine based on the presence of target in samples, however, it is not precise enough to provide accurate quantification results. Therefore, for pollutant screening, GICA is typically used as a semi-quantification tool. As a result, food and environmental monitoring technologies require a versatile and flexible method for the simultaneous detection of CBF and 3-OH-CBF that is not only sensitive, but also facile, simple in operation, and cost-effective.

Waveguide-based evanescent wave fluorescent biosensors have received wide attention due to easy miniaturization, high sensitivity, and specificity [26]. Such biosensors use the evanescent wave to excite the fluorescent dyes, which were proportional to the target. Our previous studies have proposed an integrated evanescent waveguide-based optical biosensor system for the detection of contaminants in the water environment [27,28,29,30]. To improve the light coupling efficiency, the incidence light was casted into the waveguide chip through a beveled angle to generate the reflection spot for evanescent wave-induced biosensing. By employing the competitive immunoassay, a highly reusable optical waveguide chip was fabricated via the covalent attachment of the hapten–protein conjugate, which resulted in the fluorescence-based detection of the bound fluorophore-labelled antibodies to its surface. Fluorescence was then emitted and collected by the plastic fibers located underneath the waveguide chip. The waveguide-based fluorescent biosensors have applied for small organic molecules detection, such as bisphenol A, melamine, 2,4-D, and microcystin-LR with high sensitivity and specificity [27,28,29].

To simultaneously detect CBF and 3-OH-CBF, the present study focused on developing a facile and rapid detection technology based on an optical waveguide-based fluorescent sensor and a broad-spectrum monoclonal antibody against CBF and 3-OH-CBF. To our knowledge, this is the first report on the application of an evanescent wave fluorescent biosensor to determine tCBF.

## 2. Material and Methods

### 2.1. Reagents

CBF, 3-OH-CBF, sodium dodecyl sulfate (SDS), bovine serum albumin (BSA), anhydrous N,N-dimethylformamide (DMF), N-(4-maleimidobutyryloxy) succinimide (GMBS), and 3-mercaptopropyl-trimethoxysilane (MTS) were purchased from Sigma-Aldrich Chemical Co. (St. Louis, MO, USA). Cy5.5 was obtained from GE Healthcare Life Sciences (Fairfield, USA). All analytical grade chemicals (if not specified) were utilized as received without any purification.

The broad-spectrum monoclonal antibody targeting CBF and 3-OH-CBF, simultaneously, were produced in our laboratory as described previously [24]. Labeling of the monoclonal antibody with Cy5.5 was performed as similar as the previous report [28]. Methanol was used while preparing the stock solutions of 1 g/L CBF and 3-OH-CBF (stored at 4 °C). The stock solutions were finally diluted by 10 mM phosphate-buffered saline (PBS, pH = 7.4) for further use.

### 2.2. Instrument

The waveguide-based fluorescent biosensor used in this study was described in our previous works [27,30], and its schematic diagram was shown in Figure S1. The structure of the instrument includes: optical biosensing interface, signal generation and receiving system, sample processing and flow injection system, and data processing system. The main structure of the optical sensing interface is a biochip modified with coated antigens as a reaction interface. The signal generation and reception system mainly includes lasers, optical fibers, filters and photodiodes to complete the process of fluorescence excitation and signal acquisition. The sample processing and flow injection system is responsible for automatically completing the pre-mixing of samples and antibody solutions, as well as the taking of various solutions and the discharge of waste liquids. The data processing system is mainly for the computer to digitize the collected signals.

Briefly, the 635 nm He-Ne laser (RTR Optoelectronics Technology Co., Huizhou, China) acted as the incident light. It was coupled by its beveled end into a planar waveguide. The fluorescence was released and collected through plastic fiber, which was mounted in the sensing region with a 1 mm diameter, 0.46 numerical aperture. The light was further collected after filtering through a high-pass-filter (Edmund Optics Inc., Tucson, AZ, USA) and was detected through photodiode by a lock-in enclosure amplifier.

The planar waveguide with simple geometry was adopted as our previous work [27,28]. A rectangular silica slide (60 mm in length × 15 mm in width in area and 1.5 mm in depth) with a polished 45° bevel on one endface served as the waveguide core. The other endface was black ink painted for the light absorption. Air and liquid bulk phases surround the chip served as the upper and lower cladding, respectively. According to Snell’s law, when the incident angle *θ* is higher than the critical angle *θ*_c_, the light was kept in the silica slide and propagated via total internal reflection (TIR). Assuming the refractive index of silica slide is 1.5163@632.8 nm and that of the liquid phase (water) is *n*_1_ = 1.33@632.8 nm, the calculated *θ*_c_ is equal to 61.3°. The actual incidence angle was set to be approximately 61.8° and increased by 0.5° on the basis of the critical angle of 61.3°. The length and depth of waveguide will change the light path inside the chip, and hence affect the numbers of total reflection spots formed on the surface [27]. Based on the sizes of the waveguide chip, eight individual TIR spots were formed on the surface, and anyone could be chosen for the next biosensing.

**Figure 1:**
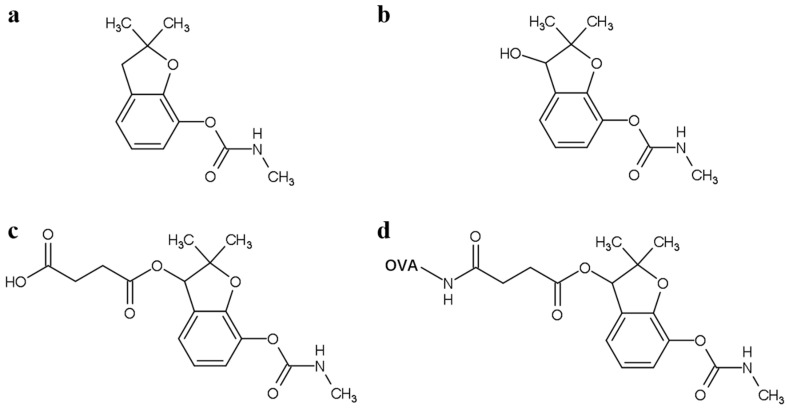
Chemical structures of the related compounds: (**a**) carbofuran, (**b**) 3-hydroxy-carbofuran, (**c**) 3-succinyl–CBF and (**d**) 3-succinyl-CBF-OVA. CBF, carbofuran; OVA, ovalbumin.

The waveguide was modified with hapten-protein conjugate with the following four steps (Figure S2). The hapten-protein conjugate is 3-succinyl-CBF-OVA (Figure 1). The waveguide was firstly immersed in piranha solution for 1~2 h to remove possible impurities and form OH group on the silica surface. The waveguide was then thoroughly cleaned by DI water and immersed into a 2% (*v*/*v*) MTS/toluene solution for another 1 h to generate sulfhydryl groups on the waveguide surface. After that, the waveguide was treated with 2 mM GMBS in ethanol for 1 h to connect the coating antigen. Afterward, 0.1 mg/mL 3-succinyl-CBF-OVA [24] in PBS (pH 7.4) solution was added dropwise to the active spot overnight at 4 °C. The waveguide was finally blocked with the isoelectric BSA solution at pH 4.6 at 4 °C overnight to reduce the non-specific adsorption [31].

### 2.3. Sensitivity and Recovery

The sensitivity was evaluated by using diluted CBF and 3-OH-CBF solutions. The analyte is diluted with 10 mM PBS buffer. 0.8 mL analyte diluted solution and 0.2 mL of 1 µg/mL Cy5.5-labeled antibody are mixed, followed by incubating at 37 °C for 5 min to leave the antibody bind with the antigen. The mixture was then pumped through the waveguide surface at the flow rate of 75 µL/min at 20 °C to make the unbound antibody to react with the surface-immobilized antigen. After that, the surface-bound antibody was finally washed using the washing buffer (0.5% SDS solution pH = 1.9) at a flow rate of 0.5 mL/min for 8 min, ready for the next testing cycle. The whole time for each cycle lasted for approximately 30 min. A four-parameter logistic equation was applied in fitting signal intensities.

$$A=\frac{A_{1}-A_{2}}{1+{\left(x/x_{0}\right)}^{p}}+A_{2}$$

where A is a biosensor signal, *x* is a concentration of CBF or 3-OH-CBF, where A_1_ and A_2_ are the top and bottom asymptotes, *x*_0_ is a concentration of CBF or 3-OH-CBF respectively at inflection, and *p* represent inflection point slope. The linear range was determined using 20% to 80% of the fluorescent intensities of the differential zone (A_1_ − A_2_). IC_50_ was identified as the signal at 50% of the signal differential region (A_1_ − A_2_). The limit of detection (LOD) was set to be 90% of the signal difference area (A_1_ − A_2_).

To evaluate the practicability of this proposed technology, a recovery study was carried out using two real water samples (tap water and river water) and two agricultural products (long bean and apple), respectively. Three different spiked concentrations (5, 10, and 20 μg/L, respectively) were tested for CBF and 3-OH-CBF, separately. The river water samples were taken from a local river near the Tsinghua campus and filtered with a 0.45 µm filter membrane to remove the particles before the test. The samples of tap water were collected from the laboratory and used directly without pretreatment. Long bean and apple were purchased from the local market, and the samples were pretreated in reference to a previously reported method [24]. A total of 5 g of pre-powdered samples were accurately weighed and spiked with various concentrations of CBF or 3-OH-CBF. The sample was homogenized with an acetonitrile of 10 mL. The mixture was extracted ultrasonically for 20 min and centrifuged for 10 min at 5000 rpm. The supernatant was extracted applying saturated solution of NaCl. The organic phase was then added to the SPE dispersion tube of QuEChERS and centrifuged for 5 min at 12,000 rpm. The supernatant was later allowed to evaporate by nitrogen blowing, and the residues were dissolved in PBS. The spiked samples were also validated by icELISA with the same sample pretreatment method. Each sample was conducted in triplicate and represented with the mean value and standard derivation (S.D.).

## 3. Results and Discussion

### 3.1. Sensing Mechanism

**Figure 2:**
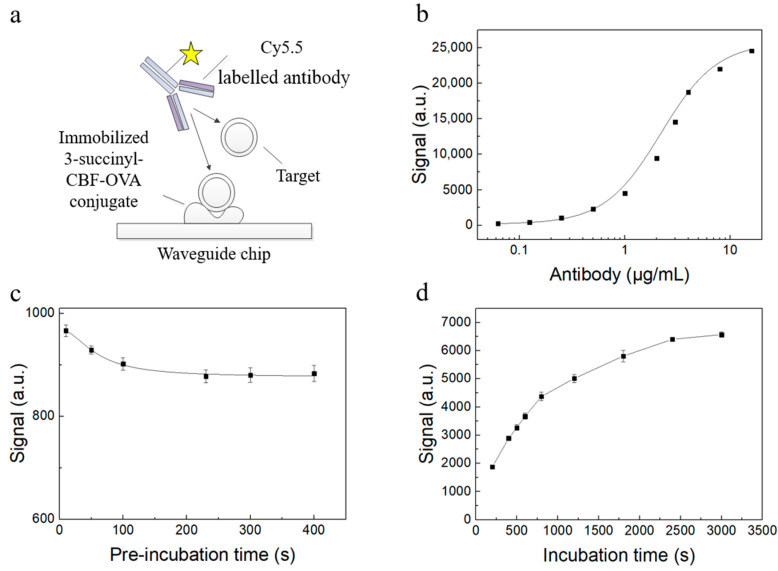
Schematic representation of the developed biosensor and its experimental optimization results. (**a**) Schematic representation of the competitive type of waveguide-based fluorescent biosensing immunoassay; relationships among the fluorescence signals and (**b**) the concentration of the Cy5.5-labeled CBF antibody, (**c**) preincubation time for 5 ng/mL CBF, and (**d**) incubation time for blank sample. Each data represents the average intensity with S.D. in triplicates.

Based on the developed waveguide-based evanescent wave fluorescent biosensor platform and surface modified waveguide chip, a competitive immunoassay was implemented (Figure 2a), which was based on the inhibition of antibody binding to the CBF immobilized on the waveguide surface in the form of 3-succinyl-CBF-OVA conjugate by the tCBF in the samples. The tested sample was firstly premixed with the Cy5.5-labelled antibody and reacted for a period of time. Then, the mixed sample was passed through the chip surface. The unbound antibody reacted with the hapten immobilized on the chip surface. Finally, the fluorescence intensity of the Cy5.5-labelled antibody attached on the chip surface was detected for the quantitative analysis. After that, the chip surface was washed by using the washing buffer to complete the regeneration of the chip for the next detection. The fluorescence intensity was positively correlated to the amount of antibody attached on the chip surface, hence inversely correlated to the concentrations of tCBF in the samples.

### 3.2. Optimization of the Experimental Conditions

Some critical parameters used for the operation of the developed immunosensor were optimized: antibody concentration, pre-reaction time, and reaction time [29]. The Cy5.5-labelled antibody concentration is essential for the detection capability of competitive immunoassay. We tried to determine the antibody concentration, which could ensure that the signal did not affect the detection accuracy with the minimum value to save the cost. As shown in Figure 2b, the antibody concentration was varied and tested separately, whereas the other conditions were the same. The curve-fitting was performed with the four-parameter logistic equation, and the linear range of signal changes with antibody concentration was 0.94–5.14 μg/L. The selected antibody concentration was 1 μg/mL, which was near to minimum value of the linear range and ensured high sensitivity with low-cost.

The effect of pre-reaction time on the signal was determined, as shown in Figure 2c. The fluorescence intensities were recorded when the pre-reaction time was prolonged, whereas the antigen concentration was held constant. Notably, the signal gradually decreased and remained almost unchanged as the pre-reaction time was longer than 240 s, indicating that the antigen in the sample fully reacted with the antibody. Therefore, the final pre-reaction time to ensure that the antigen and antibody in the sample had reacted completely was set to be 300 s.

The effect of reaction time on the signal was further determined, as shown in Figure 2d. As we expected, the fluorescent signal increased with an increase in reaction time. However, the signal growth per unit time, i.e., the slope of the curve, gradually decreased with the enlarged reaction time. When the reaction time was reached to 800 s, the slope of the curve was significantly reduced. Therefore, in consideration of the signal intensity and the measurement time, we selected 800 s as the optimal reaction time.

Under the optimized experimental conditions, we selected 1 μg/mL as the Cy5.5-labelled antibody concentration, 800 s as the reaction time, and 300 s as the pre-reaction time in the compromise of the detection time, cost, and sensitivity.

### 3.3. AFM Characterization of the Immunosensor Chip

**Figure 3:**
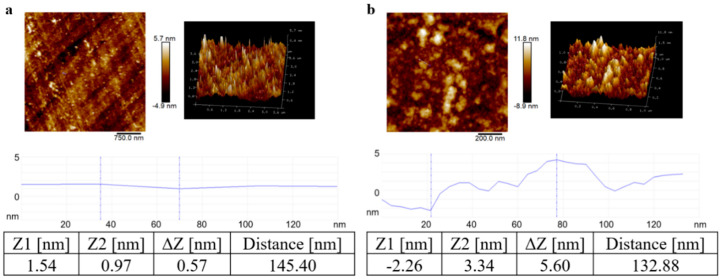
AFM topography images of chips. (**a**) Bare and (**b**) 3-succinyl-CBF-OVA conjugate-modified waveguide chips (higher right); 3-D AFM topography image (higher right) and variations in cross-section heights taken at a rough area (lower: position indicated by blue line in the topographic images).

In the optical waveguide-based fluorescent immunosensor, the main reaction and regeneration occurred in the single-layer coated antigen (3-succinyl-CBF-OVA) that was covalently bound on the chip surface. Under revealed by the AFM (Atomic Force Microscope), as shown in Figure 3, the chip surface roughness coated with 3-succinyl-CBF-OVA significantly increased compared with the bare waveguide chip. Moreover, the height of the bare chip surface was changed by 0.57 nm, and that of the 3-succinyl-CBF-OVA-coated chip was changed by 5.60 nm. Given that the Stokes radius of OVA is 2.73 nm [32], this result proved that the 3-succinyl-CBF-OVA monolayer conjugate had successfully formed on the chip surface.

### 3.4. Sensitivity

**Figure 4:**
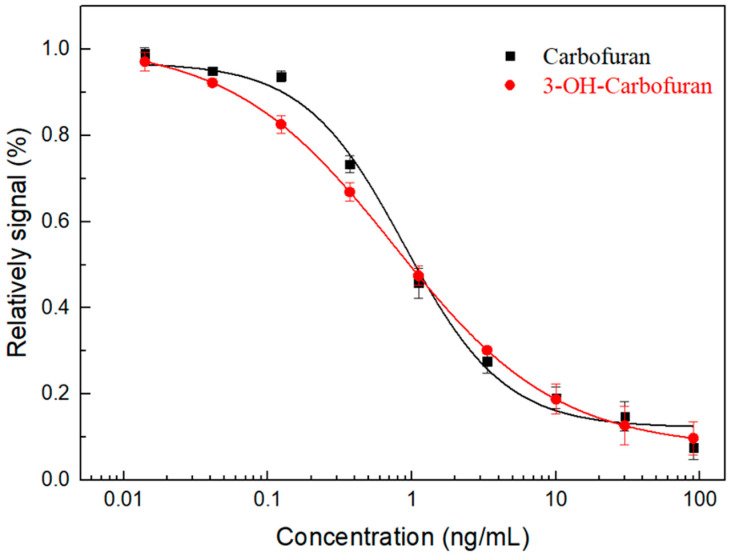
Calibration curves of the developed biosensor against CBF and 3-OH-CBF, separately. R^2^ = 0.98 for CBF and 0.99 for 3-OH-CBF, respectively. Each data represents the average intensity with S.D. in triplicates.

The standard calibration curve towards CBF and 3-OH-CBF was determined separately. As shown in Figure 4, CBF and 3-OH-CBF had linear detection ranges of 0.29–2.69 and 0.12–4.59 μg/L, IC_50_ values of 0.89 and 0.75 μg/L, and LODs of 0.13 and 0.06 μg/L, respectively. The sensitivity of this immunosensor was comparable to our previously established ELISA method [24] and highly sensitive than our previously reported colloidal gold test strip [23]. Compared with other types of CBF detection methods (Table 1), the detection ability of this method is considerable. And compared with a large number of current electrochemical CBF immunosensors, the developed fluorescent immunosensor has the ability to detect CBF and 3-OH-CBF at the same time. This biosensor-based method has outstanding advantages, such as inexpensive instruments, simplicity in operation, cost-effectiveness, and being capable of use for on-site detection.

### 3.5. Reusability

**Figure 5:**
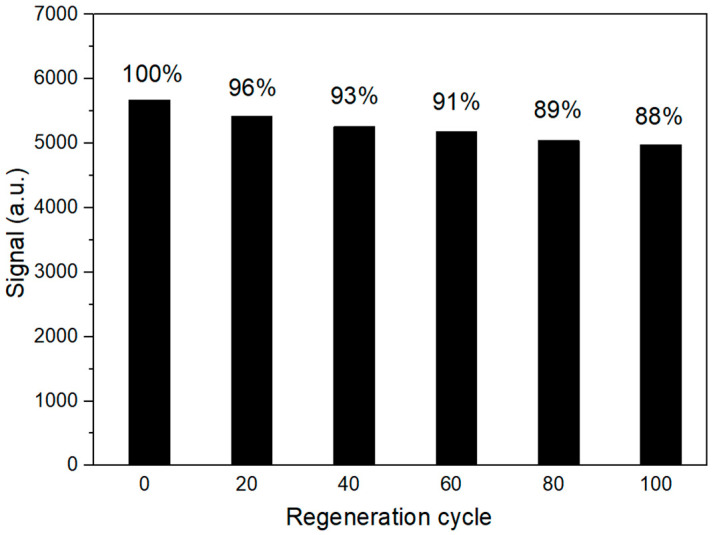
Signals recovery at each twenty regeneration cycles. The signal recovery percentages were indicated in each column.

The reusability of the biosensing chip was crucial for detection accuracy and reducing the detection cost. We recorded the detection signal towards the blank sample by using the same chip (Figure 5 and Figure S3). After 20 consecutive cycles of measurement, the signal fluctuated within a certain range. The coefficient of variation (CV) of the signal in the first 20 measurements was 2.58%. After 100 measurement cycles, the signal dropped by approximately 12%. The waveguide biosensing chip could be used for more than 100 times, confirming the excellent repeatability.

### 3.6. Recovery Study

The river water, tap water, long bean, and apple samples were collected and spiked with CBF and 3-OH-CBF separately, all samples were tested in triplicate. The recovery results are shown in Table 2. The recovery ratios of CBF and 3-OH-CBF were 98.7%–109.6% and 94.3%–104.9%, respectively. The samples were tested and validated by icELISA, as shown in Table 2, which provided a good correlation with the results determined by the immunosensor. Therefore, the immunosensor was verified to have good application potential for the detection of tCBF in water, vegetable, and fruit samples.

## 4. Conclusions

A highly sensitive broad-spectrum monoclonal antibody against CBF and 3-OH-CBF was used to establish an optical waveguide-based fluorescent immunosensor, which can enable the detections of water samples, vegetables, and fruits. The established immunosensor detection method had LODs of 0.13 and 0.06 μg/L for CBF and 3-OH-CBF, respectively, which were lower than the detection standards stipulated by most countries. The developed fluorescent immunosensor is of great significance for the simultaneous and rapid detection of CBF and 3-OH-CBF with low cost. Moreover, the chip was highly reusable, which could be reused for more than 100 times. The entire detection process lasted approximately 30 min, and was fully automated without human involvement. The good recoveries of tCBF from real water and agricultural products indicated that this technology has good application prospects in reality.

## Figures and Tables

**Table 1:** Comparison of different methods for the carbofuran detection.

Analytical Methods	Linear Range (ng/mL)	LOD (ng/mL)	References
GC	1.0–200	1.0	Petropoulou et al., 2006
HPLC	/	0.06	Lopez-Blanco et al., 2002
ELISA	0.1–1.0	/	Yao et al., 2017
GICA	/	7–10	Lan et al., 2020
Fluorimetry	4.0–560	2.0	Li et al., 2010
Amperometric immunosensor	0.1–1,000,000	0.06	Sun et al., 2012
Microcantilever-based immunosensor	0.1–1000	0.1	Dai et al., 2017
Fluorescent sensors	0.29–2.69 (CBF)	0.13	This work
	0.12–4.59 (3-OH-CBF)	0.06	

“/” means data not available in the reference.

**Table 2:** Recoveries of samples spiked with CBF and 3-OH-CBF, respectively, by using the immunosensor and icELISA. Each data represents the average intensity with standard deviation (SD) in triplicate.

**Samples**	**Spiked Value (ng/g)**	**Recovery of CBF** **%**			
		**This Method**		**icELISA**	
		**Recovery** **(%)**	**Coefficient** **Variation** **(CV) %**	**Recovery** **(%)**	**Coefficient** **Variation** **(CV) %**
River water	0	ND ^a^		ND	
	5	104.3	2.0	89.1	8.3
	10	103.2	1.3	93.3	5.7
	20	99.9	2.2	97	9.6
Tap water	0	ND		ND	
	5	105.6	0.7	101.2	3.6
	10	98.7	1.3	94.5	2.0
	20	98.8	0.5	103.8	5.2
Long bean	0	ND		ND	
	5	109.6	1.7	104.3	2.7
	10	101.8	1.6	102.8	1.4
	20	99.3	2.7	97.3	6.2
Apple	0	ND		ND	
	5	102.2	1.8	88.7	10.4
	10	103.8	2.1	95.4	7.4
	20	102.5	0.2	89.5	7.0
**Samples**	**Spiked Value (ng/g)**	**Recovery of 3-OH-CBF** **%**			
		**This Method**		**icELISA**	
		**Recovery** **(%)**	**Coefficient** **Variation** **(CV) %**	**Recovery** **(%)**	**Coefficient** **Variation** **(CV) %**
River water	0	ND		ND	
	5	98.7	1.9	93.4	6.3
	10	98.8	3.5	99.7	6.3
	20	104.2	3.4	98.1	6.8
Tap water	0	ND		ND	
	5	96.6	2.8	99.8	3.1
	10	101.8	2.4	96.1	2.8
	20	103.3	1.9	102.3	2.8
Long bean	0	ND		ND	
	5	94.3	1.8	103.3	8.0
	10	97.8	3.6	102	2.3
	20	99.2	1.7	99	7.8
Apple	0	ND		ND	
	5	104.9	1.0	91.3	9.0
	10	94.9	0.3	94.9	9.0
	20	96.4	1.5	92.6	4.5

^a^ ND means not detected.

## Supplementary Materials

The following are available online at https://www.mdpi.com/2079-6374/10/12/191/s1, Figure S1: Schematic diagram of the optical waveguide-based fluorescent immunosensor, Figure S2: Schematic diagram of the immobilization of the hapten conjugate 3-succinyl-CBF-OVA on the chip surface, Figure S3: Signal recovery after 20 cycles of measurements and regeneration with 0.5% SDS solution at pH = 1.9.
