# Supplementary material for: Waveguide-Based Fluorescent Immunosensor for the Simultaneous Detection of Carbofuran and 3-Hydroxy-Carbofuran
Source: Biosensors (Basel). 2020 Nov 27;10(12):191. doi: 10.3390/bios10120191 (PMC7761426; doi:10.3390/bios10120191)
Supplement: Supplementary file 1 [file biosensors-10-00191-s001.pdf]

biosensors

MDPI

Letter

Waveguide-Based Fluorescent Immunosensor for the Simultaneous Detection of Carbofuran and 3-Hydroxy-Carbofuran

Weiming Sun [1,2,3], Lanhua Liu [3], Abdul Ghaffar Memon [3,4], Xiaohong Zhou [3,*] and Hongwei Zhao [1,2,*]

[1] Key Laboratory of A&F Environmental Processes and Ecological Regulation of Hainan Province, College of Ecology and Environment, Hainan University, Haikou 570228, China; 18095131210068@hainanu.edu.cn
[2] State Key Laboratory of Marine Resource Utilization in South China Sea, Hainan University, Haikou 570228, China
[3] State Key Joint Laboratory of ESPC, School of Environment, Tsinghua University, Beijing 100084, China; llh1987@mail.tsinghua.edu.cn (L.L.); abdulghaffar@neduet.edu.cn (A.G.M.)
[4] Department of Environmental Engineering, NED University of Engineering and Technology, Karachi 75270, Pakistan
* Correspondence: hwzhao@hainanu.edu.cn (H.Z.); xhzhou@mail.tsinghua.edu.cn (X.Z.)

Received: 30 October 2020; Accepted: 21 November 2020; Published: 27 November 2020

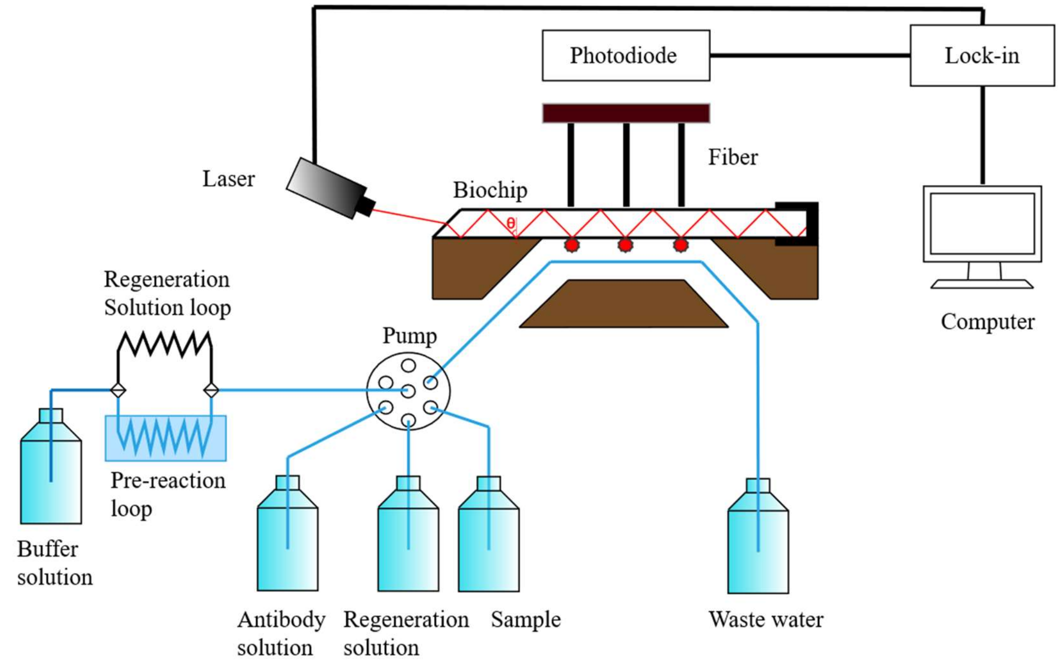

Figure S1 Schematic diagram of the optical waveguide-based fluorescent immunosensor

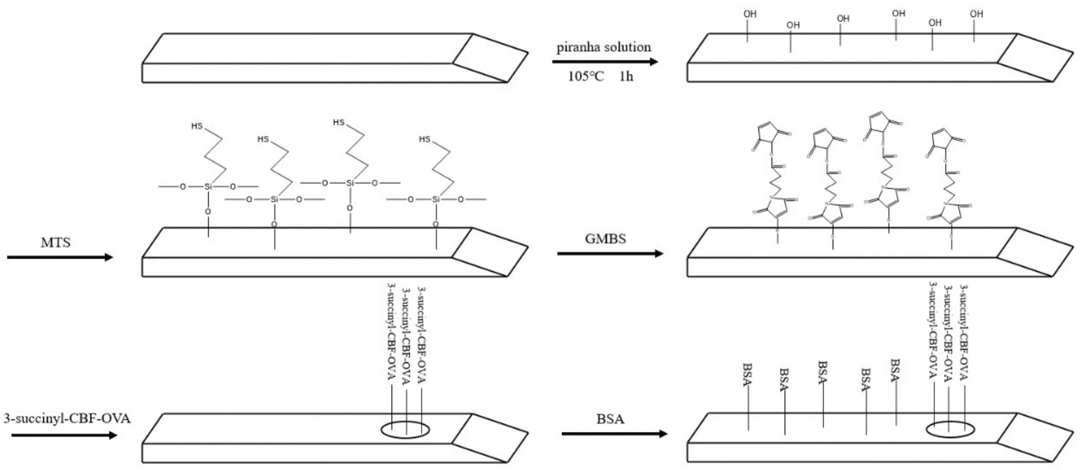

Figure S2 Schematic diagram of the immobilization of the hapten conjugate 3-succinyl-CBF-OVA on the chip surface

Antibody labeling

The antibody was labeled with the dye Cy5.5 according to the method previously described by Mujumdar et al. (1996) with a slight modification. In short, take 1 mg/mL antibody

solution and adjust the pH to 9.0–9.3. Weigh Cy5.5 according to a certain ratio (antibody/Cy5.5 1:15), and mix antibody and Cy5.5. The mixture was mixed well and reacted at 37°C for 2 hours. Dialysis was performed twice in 10mM PBS for 4 h each time to remove unbound Cy5.5. After aliquoting the obtained soultion, it was stored at -20°C.

Buffer formulation

Distilled deionized water was used throughout the investigation. Standard concentrations of the analyte were prepared from the stock solution through serial dilutions with 10 mM phosphate-buffered saline (10 mM PBS, pH 7.4). The regeneration eluent was 0.5% SDS solution (pH = 1.9). The antibody diluent comprised 10 mM PBS, 0.3% enzyme stabilizer, 0.5% thimerosal, and 0.5% BSA.

Isoelectric BSA blocking solution: 2 mg/mL BSA dissolved in 0.1 M sodium citrate/citric acid buffer (pH 4.6).

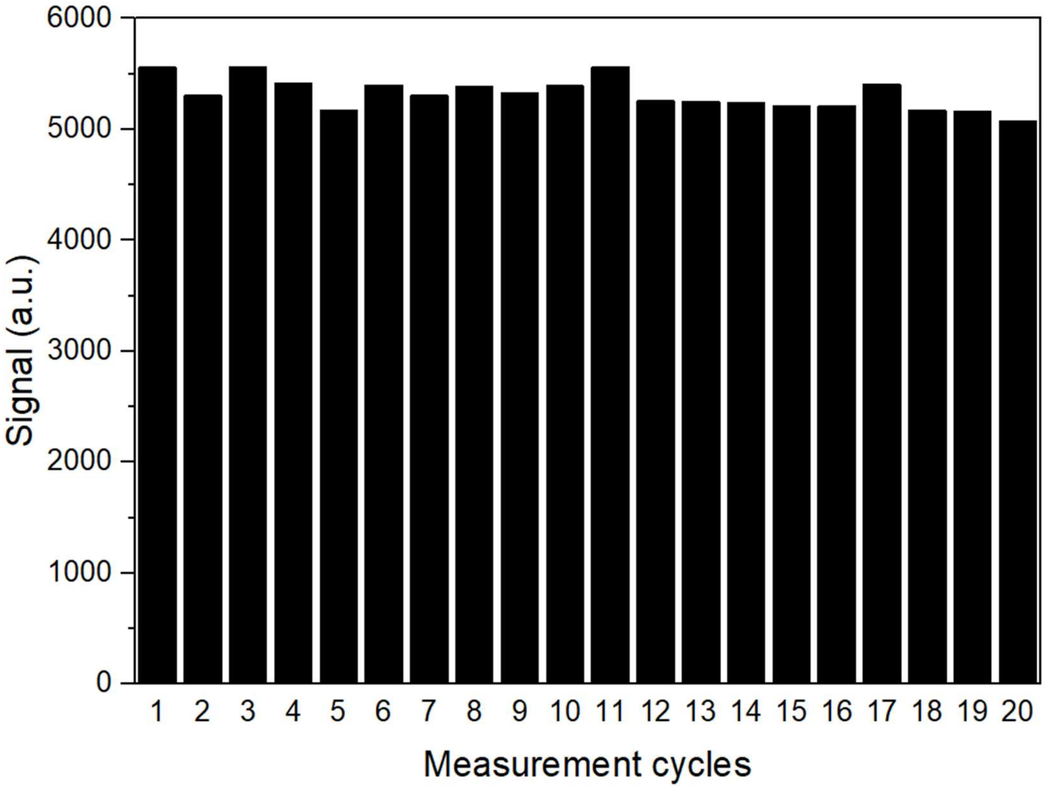

Figure S3 Signal recovery after 20 cycles of measurements and regeneration with 0.5% SDS solution at pH = 1.9
